# TBI surveillance using the common data elements for traumatic brain injury: a population study

**DOI:** 10.1186/1865-1380-6-5

**Published:** 2013-02-27

**Authors:** Latha Ganti Stead, Aakash N Bodhit, Pratik Shashikant Patel, Yasamin Daneshvar, Keith R Peters, Anna Mazzuoccolo, Sudeep Kuchibhotla, Christa Pulvino, Kelsey Hatchitt, Lawrence Lottenberg, Marie-Carmelle Elie-Turenne, Robyn M Hoelle, Abhijna Vedula, Andrea Gabrielli, Bayard D Miller, John H Slish, Michael Falgiani, Tricia Falgiani, J Adrian Tyndall

**Affiliations:** 1 Center for Brain Injury Research and Education, University of Florida College of Medicine, Gainesville, FL, USA; 2 Departments of Emergency Medicine, University of Florida College of Medicine, Gainesville, FL, USA; 3 Departments of Acute Care Surgery, University of Florida College of Medicine, Gainesville, FL, USA; 4 Departments of Anesthesiology, University of Florida College of Medicine, Gainesville, FL, USA; 5 Departments of Neurology, University of Florida College of Medicine, Gainesville, FL, USA; 6 Departments of Neurological Surgery, University of Florida College of Medicine, Gainesville, FL, USA; 7 Departments of Radiology, University of Florida College of Medicine, Gainesville, FL, USA

## Abstract

**Background:**

To characterize the patterns of presentation of adults with head injury to the Emergency Department.

**Methods:**

This is a cohort study that sought to collect injury and outcome variables with the goal of characterizing the very early natural history of traumatic brain injury in adults. This IRB-approved project was conducted in collaboration with our Institution’s Center for Translational Science Institute. Data were entered in REDCap, a secure database. Statistical analyses were performed using JMP 10.0 pro for Windows.

**Results:**

The cohort consisted of 2,394 adults, with 40% being women and 79% Caucasian. The most common mechanism was fall (47%) followed by motor vehicle collision (MVC) (36%). Patients sustaining an MVC were significantly younger than those whose head injury was secondary to a fall (*P* < 0.0001). Ninety-one percent had CT imaging; hemorrhage was significantly more likely with worse severity as measured by the Glasgow Coma Score (chi-square, *P* < 0.0001). Forty-four percent were admitted to the hospital, with half requiring ICU admission. In-hospital death was observed in 5.4%, while neurosurgical intervention was required in 8%. For all outcomes, worse TBI severity per GCS was significantly associated with worse outcomes (logistic regression, *P* < 0.0001, adjusted for age).

**Conclusion:**

These cohort data highlight the burden of TBI in the Emergency Department and provide important demographic trends for further research.

## Background

Each year, on average, traumatic brain injuries (TBIs) are associated with an estimated 1.35 million emergency department visits, 275,000 hospitalizations, and 52,000 deaths in the US [1]. This does not account for those who sustain a head injury and receive no care. TBI is a contributing factor to a third (30.5%) of all injury-related deaths in the US [1,2]. Roughly three quarters of TBIs that occur each year are concussions or other forms of mild TBI [3]. TBI is a significant burden to our health-care system. Direct medical costs and indirect costs such as lost productivity of TBI totaled an estimated $76.5 billion in the US in 2000 [4].

Traumatic brain injury is an important public health problem in the US. It is frequently referred to as the “silent epidemic” because the complications from TBI, such as changes affecting thinking, sensation, language, or emotions, may not be readily apparent. In addition, awareness about TBI among the general public is limited [1].

The CDC states: “population-based data on TBI are critical to understanding its impact on the American people. Knowing who is affected by TBIs and how they occur can help shape prevention strategies, priorities for research, and also support the need for services among individuals living with TBI” [3]. This study was undertaken specifically to address this need on a local level. By understanding our population’s patterns of injury and outcomes, we would be able to design an optimal system not only to provide acute care, but also to design programs that address research and education needs. The objective of this study was to characterize the population of patients who sustain head injuries and present to our emergency department. The reason was to understand our local demographics in an effort to design processes and interventions that would mitigate the morbidity and mortality associated with this injury pattern.

In addition to the paucity of acute epidemiological data on TBI, another problem is the variability in terms of information collected, making meta-analyses or comparisons across studies challenging. To address this, the American Congress of Rehabilitation Medicine developed a working group to delineate common data elements for demographics and clinical assessment in traumatic brain injury [5]. The current study based its data collection variables on these recommendations.

## Methods

This was a retrospective chart review that spanned a 30-month period from 1 January 2008 to 31 August 2010. Methodology and other study details are reported in accordance with STROBE guidelines [6].

The study was conducted in the Emergency Department (ED) of a level-one trauma center in the southeastern US. Our ED sees over 79,000 visits per year, and is home to both emergency medicine and general surgery residency training programs. This study was approved by our institution’s IRB as an expedited study with an HIPAA waiver.

Data were abstracted from the electronic medical record using an *a priori* designed data abstraction form. Persons entering the data were blinded to the study hypotheses and outcomes. Data were entered into our Clinical and Translational Science Institute’s REDCap database. REDCap (Research Electronic Data Capture) is a secure, Web-based application designed to support traditional case report form data capture. Statistical analyses were performed using JMP Pro10.0 for Windows. Normally distributed variables are summarized using means and standard deviations, while skewed variables are reported using medians and interquartile ranges (IQR). Missing data were recorded as unknown.

Subjects were considered eligible if they were age 18 or older and sustained a head injury, as determined by having a corresponding ICD-9 code among one of their top ten emergency department or inpatient discharge diagnoses. The codes used were 800.0-804.9, 850.0-854.1, 959.01, and 995.55 (Table 1), based on the Centers for Disease Control guidelines [7,8]. The codes were selected in order to capture all possible head injuries. If on review a record was determined *not* to have sustained a head injury, then a second member of the research team reviewed the record. If there was agreement by both researchers that the subject should not be included, they were excluded after documenting the reason. Where there was disagreement, the primary author resolved the issue via consensus.

**Table 1:** ICD-9 code

	**Corresponding TBI diagnosis**
800.00 to 804.9	Fracture of skull
850	Concussion
851	Cerebral laceration and contusion
852	Subarachnoid, subdural, and extradural hemorrhage after injury
853	Other unspecified intracranial hemorrhage after injury
854	Intracranial injury of other and unspecified nature
959.01	Other unspecified injury to head

Severity of head injury was classified using the Glasgow Coma Scale [9], with GCS 13–15 considered as mild, GCS 9–12 as moderate, and a score less than 9 classified as severe. Post-injury symptomatology collected included the occurrence of loss of consciousness (LOC), the duration of LOC, an alteration in consciousness (AOC), posttraumatic amnesia (PTA), seizure, and vomiting. An AOC was considered to be present if the patient reported any of the following: feeling dazed or confused, having difficulty thinking, or if the neurologic exam revealed a decreased mental status.

Data were also collected for mechanism of injury, including a fall, traffic accident, recreational activity, sports, and assault. Recreational activities included injuries related to bicycles, motorcycles, all-terrain vehicles (ATVs), other vehicles (e.g., scooters), watercraft, or other (horseback riding). Information about seatbelt use in people involved in traffic accidents and helmet use in people with recreational vehicle injuries was also collected.

## Results

### Demographics

**Figure 1:**
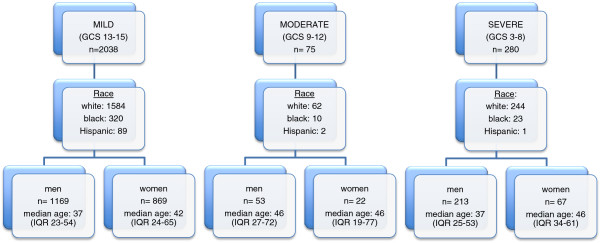
Demographic characteristics of cohort.

The cohort consisted of 2,394 subjects. The median age was 39 years, IQR 24–59. Sixty-one percent of the cohort was single, 27% married, 7% divorced, and 5% did not report a marital status. Overall, 34% of the cohort was employed, and 14% people were retired, 7.8% were students, and 17.8% were unemployed (employment status for the remaining 28% was not reported). Males accounted for 60% of the total population. The racial composition in the context of the city and county population is summarized in Table 2. The gender, age, and race breakdown by TBI severity is depicted in Figure 1.

**Table 2:** Racial and gender composition of head injury (based on the 2010 census) in adult subjects

	**City of Gainesville**	**Alachua county**	**TBI in ED**
Total population	107,742	203,051	2,394
Caucasian (Non-Hispanic White)	60.93%	66.46%	79%
Black or African-American	19.44%	17.73%	14.8%
Hispanic or Latino	10.08%	8.24%	4.3%
Asian	7.14%	5.52%	1.04%
Other Native American or Native Alaskan, Pacific Islander, two or more races, or some other race	2.41%	2.05%	1%
Female	51.46%	52.1%	40%

### Injury characteristics

The majority (88%) of the injuries occurred less than 12 h prior to presentation. Another 5% presented within 24 h. Seventy-five percent of the cohort was transported by EMS for their head injury (65.7% ground, 9.5% air). The remaining 25% presented to the ED by car, another vehicle, or walked-in. The majority (85.1%) had mild TBI (GCS 13–15); 3.1% were moderate (GCS 9–12), and 11.7% were severe (GCS 8 or below).

Loss of consciousness (LOC) was definitively reported by 51.3%, while in an additional 14.1% of subjects, it was unknown whether they lost consciousness or not. Among those with loss of consciousness, the duration of LOC was known for 59% (46.1% had LOC for 0–30 min, and 12.9% reported LOC of more than 30 min). Altered mental status was experienced by 28.9% of subjects, while 25.6% experienced post-traumatic amnesia for events before and/ or after the injury. Vomiting and seizure were less common symptoms after a TBI. In our population, 6.5% had at least one episode of vomiting after injury; 3.5% suffered from seizure after head trauma.

### Mechanism of injury

The most common mechanism for head injury for our population was a fall (46.8%). This included falls from the ground level or from any height, including trees and roofs, collapse due to syncope, and also falls associated with assault or fall by any other reason.

The second most common mechanism was motor vehicle collision (MVC), at 35.5%. Most often the subject was in the driver’s seat (61.2%), followed by front passenger seat (13%). Out of those involved in an MVC, just 46% reported wearing the seatbelt. The MVC mechanism was significantly more common in severe compared to mild TBI (52% vs. 34%, *P* < 0.0001).

**Figure 2:**
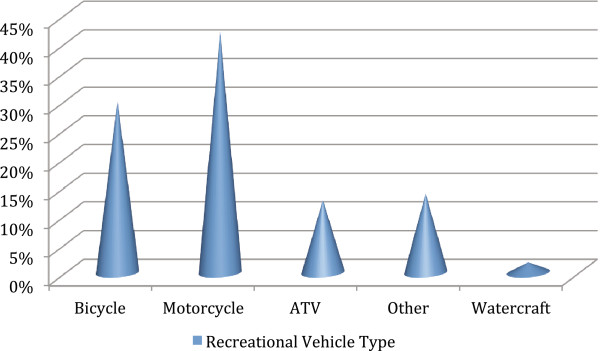
**Types of recreational vehicles involved in head injury.** Recreational vehicles were involved in 19% of head injury cases.

The next most common mechanism involved recreational vehicles, at 19%. The types of recreational vehicles are summarized in Figure 2. Of those riding a bicycle, motorcycle, or ATV, only 25.5% were wearing a helmet. Of note, the state of Florida does not have a helmet law for bicycle or ATV riders for persons over the age of 16 years [10].

### Imaging

A computed tomography (CT) scan was performed in 2201 patients, or 91.9% of the cohort. The CT was abnormal in 1,047 patients (47.6%). Excluding extracalvarial soft tissue swelling, the CT scan was abnormal in 28.4% of the cohort. The abnormalities are detailed in Table 3, and Table 4 stratifies them according to TBI severity.

**Table 3:** The percentages of head CT abnormalities

**Abnormal CT finding**	***N***	**% of total abnormal CTs (*n *= 1,047)**	**% of total CTs (*n *= 2,201)**	**% of total population (*n *= 2,394)**
Extracalvarial soft tissue swelling	368	35.1%	16.7%	15.3%
Fracture of skull	252	24.1%	11.4%	10.5%
Fracture of maxillofacial bones (except nose)	243	23.2%	11.0%	10.2%
Fracture of nasal bones	93	8.9%	4.2%	3.9%
Calvarial fracture through carotid canal	10	1%	0.5%	0.4%
Calvarial fracture through foramen magnum	8	0.8%	0.4%	0.3%
Subfalcine herniation	93	8.9%	4.2%	3.9%
Upward transtentorial herniation	8	0.8%	0.4%	0.3%
Downward transtentorial herniation	76	7.3%	3.5%	3.2%
Uncal herniation	27	2.6%	1.2%	1.1%
Tonsillar herniation	15	1.4%	0.7%	0.6%
Epidural hematoma	77	7.4%	3.5%	3.2%
Subdural hematoma	337	32.2%	15.3%	14.1%
Subarachnoid hemorrhage	319	30.5%	14.5%	13.3%
Intraventricular hemorrhage	65	6.2%	3.0%	2.7%
Parenchymal or hemorrhagic contusion	365	34.9%	16.6%	15.2%
Diffuse axonal injury (unilateral/bilateral)	50	4.8%	2.3%	2.1%
ANY bleed	701	67%	31.8%	29.8%
ANY fracture	425	40.6%	19.3%	17.8%

*All abnormalities were significantly more common with worse TBI severity* (*P* <*0*.*0001*, *ANOVA*).

**Table 4:** **Percentages based on whole cohort, *n *= 2,394**

	**Any CT abnormality as listed in table**3	**Bleed**	**Fracture**
Mild (*n* = 2,038)	739 (36%)	437 (21%)	262 (13%)
Moderate (*n* = 75)	53 (71%)	41 (55%)	22 (29%)
Severe (*n* = 280)	255 (91%)	223 (80%)	141 (50%)

### Outcomes

About 56.1% of subjects were discharged directly from the ED. Of those who were admitted, over half (51%) ended up with an ICU (intensive care unit) stay, and these were predominantly those with moderate or severe injury. The median hospital length of stay was 2 days (IQR 1–7). The median ICU length of stay was 2 days (IQR 1–10). A total of 5.4% expired in the hospital. Neurosurgical intervention including ventriculostomy, craniotomy, and craniectomy was performed in 8%. Table 5 shows the breakdown by severity; for all measures, greater TBI severity was significantly associated with worse outcomes (regression analysis, *P* < 0.0001, adjusted for age). Table 6 shows discharge dispositions stratified by severity. One would assume that none of the mild TBI patients would be admitted to the ICU, while all of the moderate and severe TBI patients would be admitted to the ICU. Our data show some deviation from this, so the specific reasons were explored. The 11.7% of patients with mild TBI who were admitted to the ICU all had either a skull fracture or intracranial hemorrhage and were admitted for either observation or surgical intervention. A small percentage also had multi-trauma. Patients with moderate or severe TBI who were not admitted to the ICU included those who were intoxicated at the time of initial evaluation but subsequently sobered up and were able to be discharged and those who expired in the trauma bay of the ED itself.

**Table 5:** Hospital admission rates for TBI based on severity

**ED TBI severity (GCS)**	**Admitted ( *n *,%)**	**ICU stay (n,%)**	**Intra-hospital death (n,%)**	**Neurosurgical intervention**
Mild (2,038)	719 (35.3%)	239 (11.7%)	16 (0.8%)	78 (3.8%)
Moderate (75)	60 (80%)	48 (64%)	9 (12%)	19 (25.3%)
Severe (280)	270 (96.4%)	248 (88.6%)	105 (37.5%)	96 (34.3%)

**Table 6:** Discharge disposition according to TBI severity

**ED TBI severity (GCS)**	**Home**	**Skilled nursing facility**	**Rehabilitation facility**	**Psychiatric facility**	**Left against medical advice**	**Death in hospital**
Mild (2,038)	1,819 (89.3%)	117 (5.7%)	37 (1.8%)	11 (0.6%)	37 (1.8%)	16 (0.8%)
Moderate (75)	41 (54.7%)	11 (14.7%)	11 (14.7%)	1 (1.3%)	2 (2.6%)	9 (12%)
Severe (280)	71 (25.4%)	40 (14.3%)	59 (21.1%)	3 (1.1%)	2 (0.7%)	105 (37.5%)

Almost 5% returned to the ED within 72 h (Table 7), most commonly for symptoms of post-concussive syndrome (30%), with the predominant complaint being headache. The next most common reason for 72-h ED return was because the patient was specifically called back for a missed read on radiologic imaging (13%). A return ED visit was significantly more common in patients with mild TBI (*P* = 0.0218, chi-square), likely because higher severity patients are admitted to the hospital. Six percent were re-admitted to the hospital within 30 days. The readmission was usually unrelated to the antecedent TBI, although of the reasons specified, a chronic or worsening subdural hematoma was the culprit in 5%.

**Table 7:** The **72-h return to the ED and 30-day re-admission rates by severity**

**ED TBI severity (GCS)**	**Return to ED within 72 h ( *n *,%)**	**Re-admitted to the hospital within 30 days ( *n *,%)**
Mild (2,038)	102 (5%)	105 (5.1%)
Moderate (75)	4 (5.3%)	6 (8%)
Severe (280)	5 (1.8%)	24 (8.6%)

## Discussion

### Why the current study is important

Our findings characterize injury severity, etiology, symptomology, and outcome in patients with acute TBI presenting to a Trauma Level I teaching institution. International think tanks identified areas of focus for TBI research [11,12]. A consistent underlying theme is the need to define epidemiology and basic hospital outcomes for local populations. The current cohort provides an epidemiologic account of TBI patients that will add to understanding the magnitude of TBI, drive research priorities, and identify clinical areas of need.

Our descriptive study is unique in several ways and adds to the literature a novel description of TBI patients. The current study represents one of the largest cohorts of mild TBI patients in the civilian population. Mild TBI (mTBI) accounts for 80–85% of all TBIs and as such represents a large proportion of disability from TBI [13]. This improved understanding has led to more widespread recognition [14]. Clinicians and legislators have recognized the consequences of mild TBI can be quite significant.

### Review of other TBI cohort studies

There have been a few other cohorts describing TBI, but none have described the acute symptomatology with the level of detail that the current study does or in a population that has generalizability. Thus, a survey of soldiers deployed in Iraq [15] does report symptomology, but the cohort is not varied enough in mechanism, gender, and age to represent the general population, and it describes the symptoms experienced by the soldiers reported at a date much later than the injury, which inherently carries recall bias. A historical study from the Mayo Clinic reports on incidence morbidity and mortality outcomes after TBI for Olmsted county for the years 1935–1974, but could not comment on acute emergency department variables because of the design and time period [16]. Similarly, a New Zealand study reports on the incidence and outcomes, but did not assess acute symptomatology or clinical variables [17]. Two papers report on emergency department visits for TBI from 1992–94 [18] and 1995–16 [19]; however, these studies are not only now 15–20 years old, but also rely on data collected from the National Hospital Ambulatory Medical Care Survey [20], in which hospital staff in selected US hospitals are instructed to complete patient record forms for a systematic random sample of patient visits during a randomly assigned 4-week reporting period. This survey has inherent limitations, including a modest response rate, optional participation without incentives, and inability to capture many patient level data including acute symptomatology. More recent cohorts have been described as well, but these have been limited to moderate and severe TBI [21,22] or patients admitted to an ICU [23], both distinct from the cohort described in the current study.

### Comparing outcomes between the current study and the other published studies

Among the moderate and severe TBIs, comparisons can be made to other studies even when emergency department acute symptomatology was not specifically studied, as these are a less heterogeneous group than mild TBI patients. For example, Andriessen et al. [21] prospectively enrolled 508 moderate and severe patients across five level I trauma centers in The Netherlands. Their rates of abnormal CTs were comparable to the current cohort (56 vs. 71% for moderate and 81 vs. 91% for severe) when accounting for extracalvarial soft tissue swelling. These studies also mirror neurosurgical intervention (26% vs. 25% for moderate and 29% vs. 34% for severe).

A study examining 476 moderate and 1,701 severe TBI patients across Europe and North America [20] reported MVC as the mechanism at a rate similar to that in the current report (57 vs. 52% of the TBIs in their severe cohort were due to an MVC mechanism).

Given the lack of other large cohort studies that focus on acute symptomatology in mild TBI, comparisons of our study findings within the mild TBI cohort to other such populations are not possible. We did note, for instance, that mild TBI had unexpected amounts of pathology on CT (21% had bleeds, 13% had fractures), neurosurgical intervention rates (3.8%), and death rates (0.8%). This higher than expected acuity in this group may indicate the GCS scale was an underestimation of illness or that the medical community’s assumptions about “mild” TBI may be underestimating the disease process.

### Limitations

While some of the explicit strengths of our study include the large number of patients with all severities of TBI (especially mild TBI) presenting to our ED and the ability to collect clinical information unavailable through billing coding, there are some inherent weaknesses in our methods as well. The limitations of the current study are: (1) the cohort was assembled using billing codes as a starting point; (2) data were abstracted from medical record review and therefore certain pieces of data were missing for some subjects; (3) these data pertain to a single medical center, although the sample appears to be representative of national demographics.

## Conclusion

These cohort data highlight the burden of TBI in the Emergency Department and provide important demographic trends for further research. The higher than expected positivity as well as intervention rates of the mild TBI patients is a important finding that warrants further study.

## Competing interests

The authors declare that they have no competing interests.

## Authors’ contributions

LGS, ANB, PSP, and YD conceived the study. ANB, PSP, YD, AM, AV, SK, CP, and KH collected the data. ANB and LGS performed the statistical analyses and ANB, PSP, and YD cross checked the data. LGS and KRP supervised the conduct of the research and data collection. LGS, RMH, and ANB drafted the manuscript, and all authors contributed substantially to its revision. All authors read and approved the final manuscript.
